# Exploring and analyzing learning curves in robotic-assisted thoracoscopic anatomical lung resections: A systematic review and meta-analysis

**DOI:** 10.1016/j.xjon.2025.07.011

**Published:** 2025-07-24

**Authors:** Jade Qu, Leanne Harling

**Affiliations:** a Department of Thoracic Surgery, King's College London, London, United Kingdom; b Department of Surgery and Cancer, Imperial College London, London, United Kingdom

**Keywords:** robot-assisted surgery, learning curves, anatomical lung resection, lobectomy, segmentectomy, CUSUM

## Abstract

**Objectives:**

There has been a steady increase in the uptake of robotic-assisted thoracic surgery over recent years, with a necessary focus on training. The early learning curve has been extensively debated; however, a detailed understanding of how this extends as we gain experience has been poorly discussed. This study assesses the congruency and depth of the learning curve in robotic-assisted thoracic surgery of anatomical lung resection.

**Methods:**

All studies reporting a quantitative assessment of operator learning curve in robotic anatomical lung resection before March 1, 2024, were included. Two authors extracted data, and study quality was assessed according to Preferred Reporting Items for Systematic Reviews and Meta-Analyses guidelines. Meta-analysis was performed using random effects modeling.

**Results:**

Twenty-nine studies including 106 surgeons were identified. A triphasic learning curve was identified with “competency” achieved at 20 (interquartile range, 13) cases, and “proficiency” at 60 (interquartile range, 37.5) cases. Weighted mean difference operating time between novice (P1) and proficiency (P2) was 34.1 minutes and between “competency” (P2) and “proficiency” (P3) was 17.5 minutes. This decreased with newer generations of robotic technology (Da Vinci Xi, weighted mean difference P1 vs P3: 42.3 [22.0, 62.5] vs S/Si, 57.4 [45.6, 69.2] minutes).

**Conclusions:**

The learning curve in anatomical lung resection is triphasic, with a reproducible extension beyond the initial proficiency phase. As robotic lung resection becomes more widespread, we must better understand the translation of this learning pattern among trainee surgeons to maintain excellence in clinical outcomes while facilitating training of the next generation.

**Figure:**
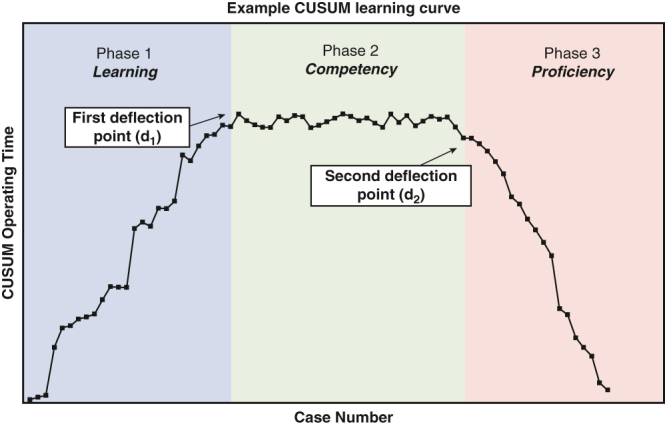
Example CUSUM phases and deflection points modeled on a single learning curve.

**Central Message**

RATS anatomical lung resection has a potential learning curve of 20 cases to reach competency and 60 cases for proficiency.

**Perspective**

Learning curves in robotic anatomical lung resections are poorly defined. Existing literature demonstrates a competency learning curve of 20 cases and a proficiency learning curve of 60 cases. However, as the demographic of learners changes, and more operating platforms become available, existing analysis fall short of a comprehensive assessment.

Robotic-assisted thoracoscopic surgery (RATS) has rapidly grown in popularity over the past 20 years.^E1^ Robotic surgical approaches have been shown to offer advantageous patient outcomes such as decreased postoperative pain, faster recovery, and decreased length of stay compared with open thoracotomy.^E2^ The technique also has inherent benefits for the surgeon, who is operating with 3-dimensional vision as well as increased degrees of freedom of movement and improved ergonomics.^E3^ In recognition of this paradigm shift, health care institutions are investing more than ever in establishing robotic services and upskilling the surgical workforce.^E4^

Until recently, lobectomy had remained the “gold standard” for early-stage lung cancer resection; however, the recent publication of the JCOG0802 and CALGB140503 trials have indicated comparable efficacy of sublobar resection with that of lobectomy.^1^^,^^2^ Although surgical approach was not the focus of either trial, it is recognized that RATS significantly facilitates complex anatomical segmentectomy and has led to its application to these procedures worldwide.^E5^

Amidst this advancement, training and upskilling the existing workforce remains a predominant rate limiting factor. We understand that learning curves can play a role in describing the rate at which proficiency is achieved. However, in the current literature, this has been poorly defined.^E6^ There is no validated approach as to how to assess a learning curve, with the focus largely on numerical outcomes such as total operating time.^E7^ Although a numerical value can be generated for the number of cases it takes to reach an assumption of “proficiency,” there lacks a better understanding of what we seek to demonstrate with these values.

As a result of early and widespread adoption of robotics in urologic procedures, the learning curve in urologic surgery has been analyzed more extensively than in other surgical disciplines.^E8^ Amidst this rapid advance, there is now a precedent of detailed learning curve analysis including integration of proficiency-based assessment scores, and procedure-specific, step-based analysis.^E9^

As the potential role of robotics in thoracic surgery expands, this necessitates a better understanding of how surgeons are learning these new surgical techniques and the associated patient outcomes. This review and meta-analysis aim to (1) explore the existing literature on reported learning curves for anatomical lung resections, (2) assess the congruency of assessment approaches and outcomes, and (3) delineate areas of further investigation or improvement.

## Methods

### Search Strategy

The methods for this systematic review were conducted in accordance with the Preferred Reporting Items for Systematic Reviews and Meta-Analyses guidelines. A systematic literature search was conducted across PubMed, Cochrane Library, and Ovid MEDLINE for full-text, English-language articles published between inception to January 2024. Key search terms included “Robot∗,” “Surg∗,” “Learning Curve,” “RATS,” “RTS,” “Lobectomy,” “Segmentectomy,” and “Anatomical Lung Resection.” The full search strategy for Ovid MEDLINE is shown in Table E1. Reference lists of the included articles were also searched for additional studies.

### Inclusion and Exclusion Criteria

Studies were considered for inclusion if they reported an operator “learning curve” measured using number of cases with a quantitative performance metric such as total operating time. Studies were excluded if they (1) were case reports without sufficient data on surgical approach, patient demographics, and overall perioperative outcomes; (2) had overlapping or duplicated data sets, in which case data published most recently were considered; and (3) lacked a specified statistical analysis detailing a point of significant difference or change across the learning curve.

### Data Extraction, Quality Appraisal, Quality Assessment

Two reviewers independently identified potentially relevant articles, and conflicts between reviewers were subsequently discussed. Attempts were made to clarify any insufficient/unclear data from authors of included studies, as required.

Data were extracted such that each study was effectively treated as a case series. If a study reported more than one surgeon's learning curve, then each data set was treated as an individual case series, available data notwithstanding. Information regarding procedures included, statistical analysis method, definition, and numerical value of the first “end point” was collected. Secondary end points of interest were alternative noncase volume learning curve metric analysis ie, docking time, console time, and perioperative outcomes such as blood loss, and length of stay. The Risk of Bias in Non-randomised Studies of Interventions Version 2 tool was used for risk of bias assessment.^E10^

### Statistics

A meta-analysis of proportions of means was performed for both categorical and continuous variables via random effects models to account for variation among the study variables. This was done using Stata (version 18.0; StataCorp).

**Figure 1:**
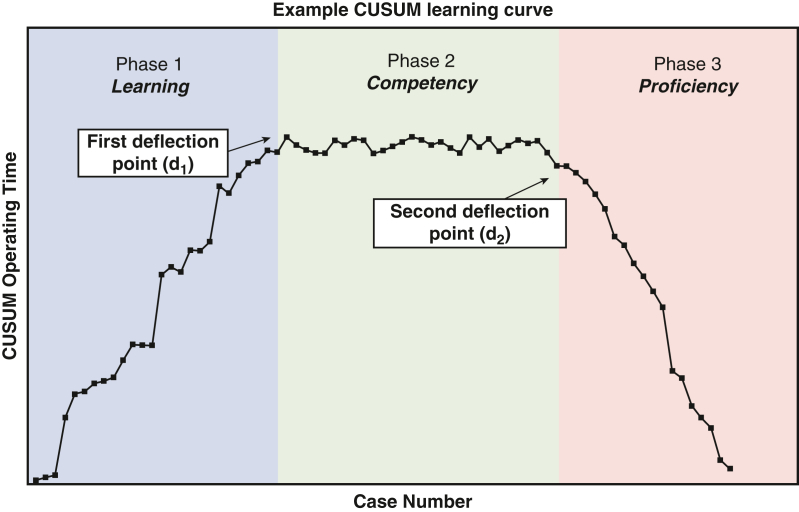
An example of a CUSUM curve across three phases, highlighting 2 key deflections, points d_1_ and d_2_. This figure serves as a visual guide only and does not depict actual data. *CUSUM*, Cumulative sum.

Averages are expressed as mean ± standard deviation or median ± interquartile range (IQR) where data are not normally distributed. These were expressed for each end point with learning curve phases 1, 2, and 3 defined according to deflection points d_1_ and d_2_, as show in Figure 1.

Data-pooling techniques, as described in the Cochrane Handbook, were used to combine the dataset.^E11^ From the pooled data, weighted mean differences (WMDs) were calculated to summaries the overall effect. Results are presented with 95% confidence intervals (CIs), and the *I*^2^ statistic, which quantifies the proportion of variability in effect estimates across studies that is attributable to heterogeneity rather than chance, was employed to evaluate heterogeneity.^E12^

Subgroup analyses were performed for operating platform generation, blood loss, and length of stay data on the basis of the availability of phase-specific data for mean operating time. WMD was also calculated across the phases for operating platform and blood loss.

## Results

**Figure 2:**
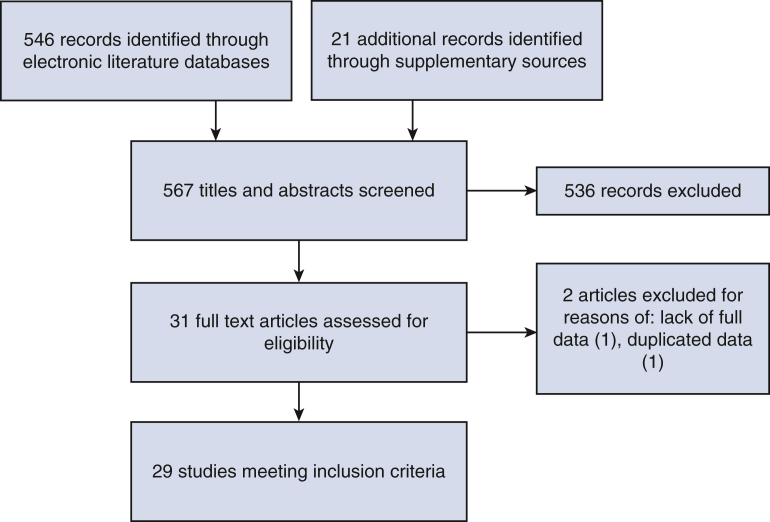
Preferred Reporting Items for Systematic Reviews and Meta-Analyses diagram of included studies.

A total of 567 articles were searched, and 29 studies met the inclusion criteria (Figure 2), encompassing a total of 107 surgeons and 9017 patients. Study duration varied with a range of 9 to 95 months. Twenty-three studies were retrospective (Table E2).^3^^,^^4^, ^5^, ^6^, ^7^, ^8^, ^9^, ^10^, ^11^, ^12^, ^13^, ^14^, ^15^, ^16^, ^17^, ^18^, ^19^, ^20^, ^21^, ^22^, ^23^, ^24^, ^25^, ^26^, ^27^, ^28^, ^29^, ^30^ The mean age of patients included was 64.7 ± 6.1 years, and 52.8% of the cohort were female.

### Baseline Demographics

Characteristics of the included studies are shown in Table E2.

### Learning Curve Analysis

Learning curve analyses were conducted using either cumulative sum (CUSUM) analysis—in which deviations from a target value are cumulatively plotted on a graph—non-CUSUM regression models, or outcome-based analyses. A total of 16 studies used the CUSUM method for learning curve analysis. Six studies used linear regression methods; the remaining 7 studies included a variety of calculated statistical significance analyses to generate a “learning curve” (Table E2). All but one^3^ used operating time as the metric analyzed to assess change. Su and colleagues^3^ analyzed learning curves on the basis of rates of prolonged air leak postlobectomy.^3^ Eighteen studies solely analyzed the learning curve on the basis of operating time.

All statistical models focused on a “deflection point” (d_1_) at which competency was assumed to have been achieved. Competency was loosely defined by an observed plateau or decrease in change in operating time (Figure 1). This generated a minimum of 2 phases within the data (before and after d_1_); however, CUSUM analysis allowed for the inclusion of a third phase postplateau, which introduced the consideration of a “proficiency” phase and accounted for the second deflection point (d_2_).^E13^ Median d_1_ was calculated to be 20 cases (IQR, 13) and median d_2_ was 60 cases (IQR, 37.5).

Nineteen studies reported mean total operating time. Meta-analysis indicated a mean of 159.6 minutes (95% CI, 134.8-184.4) with high heterogeneity (*I*^2^, 66.2%) (Table 1). Sixteen studies reported a mean phase 1 operating time of 198.3 minutes (95% CI, 173.7-222.9), with lower heterogeneity (*I*^2^, 36.3%). Sixteen studies included a mean phase 2 operating time of 160.1 minutes (95% CI, 135.3-185.0; *I*^2^, 52.5%). Calculated WMD between phase 1 and phase 2 was 34.1 minutes (95% CI, 25.7-42.3; *I*^2^, 64.6%) (Table 2).

**Table 1:** Meta-analysis of reported overall and phase-specific operating time, blood loss, and length of stay

Outcome measures	Phase 1	Phase 2	Phase 3	Total
Operating time, min	198.3 (173.7-222.9) *P* < .073	160.1 (135.3-185.0) *P* < .007	126.9 (107.9-146.0) *P* < .340	159.6 (134.8-184.4) *P* < .0001
Blood loss, mL	68.9 (33.6-104.2) *P* < .034	48.7 (18.2-79.2) *P* < .0001	60.6 (7.5-113.7) *P* < .0001	47.4 (21.8-73.0) *P* < .0001
Length of stay, d	4.4 (3.7-5.1) *P* < .881	4.6 (3.8-5.2) *P* < .974	4.2 (3.5-4.9) *P* < .929	5.1 (3.6-6.5) *P* < .998

Values in parentheses are 95% confidence intervals.

**Table 2:** Weighted mean difference between phase-specific operating time data and intraoperative blood loss data

Outcome measures	Phase 1 vs phase 2	Phase 2 vs phase 3	Phase 1 vs phase 3
Operating time, min	34.1 (25.7-42.3) *P* < .004	17.5 (10.2-24.8) *P* < .0001	51.4 (42.0-60.7) *P* < .0001
Blood loss, mL	16.1 (2.7-29.4) *P* < .0001	6.0 (−9.0 to 21.0) *P* < .0001	28.3 (−4.9 to 61.4) *P* < .0001

Values in parentheses are 95% confidence intervals.

Nine studies reported a mean phase 3 operating time of 126.9 minutes (95% CI, 107.9-146.0; *I*^2^, 11.4%) (Table 1). The WMD between phase 1 and phase 3 was 51.4 minutes (95% CI, 42.0-60,7; *I*^2^, 77.9%), whereas between phase 2 and phase 3, this was reduced to 17.5 minutes (95% CI, 10.2-24.8; *I*^2^, 79.1%) (Table 2). Nine studies failed to specify which robot was used; within those, 4 studies only mentioned the use of a da Vinci robot but not which generation (Table E2). Operating time data from both the S/Si group and Xi group indicated the same overall decreasing trend across all 3 phases. Although overall operating time reported was similar at approximately 150 minutes; by phase 3, the Xi cohort was approximately 20 minutes less than the S/Si cohort (120.1 vs 140.3 minutes) (Table 3). WMD calculated across the phases indicated similar differences between phase 1 versus phase 3, and phase 2 versus phase 3 in both generations (Table 4).

**Table 3:** da Vinci platform generation on the basis of meta-analysis of phase-specific mean operating times

Operating platform	Phase 1	Phase 2	Phase 3	Total
Da Vinci S/Si, min	205.6 (166.2-245.1) *P* < .017	167.9 (125.2-210.5) *P* < .001	140.3 (119.3-161.3) *P* < .533	152.1 (128.5-175.7) *P* < .537
Da Vinci Xi, min	184.1 (148.8-219.4) *P* < .473	144.6 (115.3-174.0) *P* < .788	120.1 (80.6-159.6) *P* < .717	148.3 (111.5-185.2) *P* < .065

Values in parentheses are 95% confidence intervals.

**Table 4:** Weighted mean difference between platform generation-specific and phase-specific operating times

Operating platform	Phase 1 vs phase 2	Phase 2 vs phase 3	Phase 1 vs phase 3
Da Vinci S/Si, min	40.4 (31.9-48.8) *P* < .182	17.4 (7.4-27.5) *P* < .015	57.4 (45.6-69.2) *P* < .012
Da Vinci Xi, min	120.1 (80.6-159.6) *P* < .717	16.6 (−1.7 to 34.7) *P* < .0001	42.3 (22.0-62.5) *P* < .001

Values in parentheses are 95% confidence intervals.

Of the included studies, 17 focused exclusively on lobectomy data, whereas 4 analyzed only segmentectomy data. Subgroup analysis, stratifying results by procedure type, revealed a general decrease in operating time across phases for both procedures. However, lobectomy consistently required longer operating times compared with segmentectomy, with a difference of approximately 55 minutes for overall operating time (Table 5). WMD analysis demonstrated more pronounced changes across learning curve phases for lobectomy procedures (Table 6). Eight studies reported postoperative blood loss outcomes. Mean total blood loss was 47.4 mL (95% CI, 21.8-73.0; *I*^2^, 72.9). Eleven studies reported mean blood loss during phase 1 of 68.9 mL (95% CI, 33.6-104.2; *I*^2^, 48.8%). Eleven studies reported mean blood loss during phase 2 of 48.7 mL (95% CI, 18.2-79.2; *I*^2^, 70.2%). Nine studies reported mean blood loss during phase 3 of 60.6 mL (95% CI, 7.5-113.7; *I*^2^, 93.9) (Table 1). On the basis of this, the WMD between phase 1 and phase 2 was 16.07 mL (95% CI, 2.7-29.4; *I*^2^, 75.7%), between phase 2 and 3 WMD was 6.0 mL (95% CI, −9.0 to 21.0; *I*^2^, 96.5%), and between phase 1 and phase 3 was 28.3 mL (95% CI, −4.9 to 61.4; *I*^2^, 96.3%) (Table 2). Length of stay was approximately at 4 days across all these phases, 4.4 (95% CI, 3.7-5.1) in phase 1, 4.6 (95% CI, 3.8-5.2) in phase 2, and 5.1 (95% CI, 3.6-6.5) in phase 3. Both blood loss and length of stay data demonstrated varying degrees of decreasing trends across the 3 phases.

**Table 5:** Meta-analysis of phase specific segmentectomy versus lobectomy specific operating time data

Resection type	Phase 1	Phase 2	Phase 3	Total
Segmentectomy, min	174.8 (155.2-194.5) *P* < .393	123.9 (97.7-150.0) *P* < .356	115.6 (73.6-157.6) *P* < .055	126.5 (109.5-143.5) *P* < .521
Lobectomy, min	233.8 (206.2-261.4) *P* < .725	180.7 (146.9-214.5) *P* < .038	154.1 (116.1-192.1) *P* < .640	181.1 (156.5-205.7) *P* < .954

Values in parentheses are 95% confidence intervals.

**Table 6:** Weighted mean difference between procedure-specific and phase-specific operating times

Resection type	Phase 1 vs phase 2	Phase 1 vs phase 3	Phase 2 vs phase 3
Segmentectomy, min	39.0 (32.7-45.3) *P* < .419	45.7 (40.7-50.6) *P* < .420	12.1 (−2.2 to 26.4) *P* < .002
Lobectomy, min	40.8 (29.6-52.0) *P* < .185	59.4 (49.3-69.6) *P* < .259	19.5 (8.6-30.4) *P* < .019

Values in parentheses are 95% confidence intervals.

## Discussion

The length of the lobectomy-only learning curve has been reported to be between 20 and 26 cases in previous analyses.^4^^,^^31^ This is similar to the results in our meta-analysis, demonstrating a learning curve of 20 cases with considerable heterogeneity. This was supported by a significant decrease in weighted mean operating time between phases 1 and 2, indicating a clear deflection point at approximately 25 cases.

Because anatomical lung resection forms the bulk of thoracic cancer care surgical caseloads,^E14^ it is important to consider whether the reported 25-case learning curve for RATS is robust and how this relates to procedural and clinical outcomes. The European Board of Thoracic Surgery requires evidence of at least 100 cases for completion of surgical training,^32^ whereas video-assisted thoracic surgery (the current standard for lobectomy) has a long established learning curve of 50 cases.^E15^^,^^E16^ These benchmarks are 2 to 4 times greater than the reported RATS learning curve.

Existing literature, such as Wilson-Smith et al,^31^ primarily focused on the initial phase of the CUSUM curve, where operating times increase cumulatively as surgeons familiarize themselves with the platform until a plateau is reached. This method relies on a predetermined target value to calculate cumulative progress as seen in Equation (1):

(1) $${CUSUM}_{i}=\sum_{\kappa =1}^{i}\left(x_{\kappa }-\mu \right)$$

Where CUSUM_*i*_ is the cumulative sum score of the *ith* procedure. $x_{\kappa }$ is the mean console time of the *jth* procedure, and μ represents the overall mean of $x_{\kappa }$ in each cohort.

Detailed analysis, however, revealed a second deflection point (d_2_) after the plateau; highlighting a potential third phase of learning (Figure 1). This third phase models a downward trend in mean operating time and hence a potential “proficiency” phase achieved at approximately 60 cases. Because CUSUM is a cumulative measure, a downward trend reflects a decrease in operating time relative to the target value, demonstrating the process approaching maximum efficiency with minimal deviations from the target performance. Nevertheless, the calculated IQR of 37 indicates substantial variability in the data. Without information on case complexity, it's challenging to identify the specific factors influencing individual learning curves. It remains uncertain at which point across these 3 phases a surgeon can be formally deemed proficient. However, when analyzed across specific learning phases, other perioperative and intraoperative outcomes have emerged as significant points of interest and highlight the need to include data beyond operating time.

MASTERY (Measuring the quality of surgical care and setting benchmarks for training using Intuitive Data Recorder technology) is an in-progress study attempting to establish specialty and procedure-specific performance benchmarks.^33^ One interesting convergence point between this meta-analysis and the MASTERY study would be where these benchmarks fall relative to the 2 deflection points when comparing mean operating times.

However, these thresholds are purely objective, numerical outcomes, and none of the included studies employed a proficiency scoring system. Proficiency scores such as the Global Evaluative Assessment of Robotic Skills score, often used in simulation-based training, provide a broader evaluation of robotic skills beyond just speed or task completion.^34^ This can offer valuable context as it is possible to achieve a specific time-based benchmark without adequate control or efficiency. It is important that assessment of learning also includes evaluation of overall surgical performance intra-operatively.

### Platform Agnostic or Platform Specific?

As of 2024, only 2 companies are CE-marked and approved for RATS in the United Kingdom: the CMR Versius and Intuitive da Vinci.^35^ The Versius device was only approved for thoracic surgery in 2022,^36^ 2 decades after the first da Vinci device received approval from the Food and Drug Administration.^37^ The majority of data in this analysis is historical and therefore the only platforms represented are the da Vinci S, Si, X, and Xi.

There is no standardization among robotics platforms, and there are fundamental differences between the CMR and Intuitive devices. Versius is designed to be modular, portable and does not use foot pedals.^38^ These distinctions result in different ergonomics, and the 2 platforms cannot be assumed to be comparable.

It is undeniable that Intuitive's market dominance has skewed the data toward a da Vinci−specific learning curve. There is currently no thoracic dataset available for the CMR Versius, although preliminary data was offered at the Society of Cardiothoracic Surgery Annual Meeting 2024 indicating a shift in the status quo.^39^ In anticipation of this change, initiatives such as the ROBO-start program at Guy's St Thomas NHS Foundation Trust have started offering early robotic multiplatform learning.^40^

We must be mindful of the ever-changing dynamics of who the learner is and what they are learning as we attempt to integrate robotics into the training pathway. As more robotic platforms will inevitably enter the market, training and research must place a greater emphasis on platform-agnostic approaches to accommodate this.

### Limitations

Most studies were retrospective, involving single institutions and single-surgeon cases. There was no reported data on those who were not able to overcome the learning curve. There is an inherent publication bias, because it is possible that surgeons who found the technique difficult never fully integrated robotics into their practice and hence did not report the caseload required to realize this; data may not have been included for those dropout data points. Most studies included analyzed senior surgeons with extensive open thoracotomy and video-assisted thoracic surgery experience who have already achieved these thresholds. This offers little insight into how the learning curve is affected by having to learn both robotic techniques and basic thoracic operative knowledge. Hence, we may be observing a shortened learning curve. Furthermore, lack of information on specific caseloads makes it difficult to evaluate how more complex cases may prolong what otherwise could be a shorter third phase of learning. The literature also tends to neglect the influence of the wider surgical team, including the bedside assistant and theater staff, on operative outcomes. Moreover, most studies report mean operative times, which are vulnerable to distortion by outliers, whereas the use of CUSUM as a metric introduces variability and potential misinterpretation because of its self-referential characteristics.^E17^

## Conclusions

As robotics become more commonplace in operating theaters, those undertaking robotics training will inevitably be earlier in their surgical training pathway. Our review of the literature suggests a potential 3-phase learning model for robotic anatomical lung resection. However, how these phases translate into true proficiency remains unclear, as does the influence of previous surgical experience and varying robotic platforms. Future research should prospectively record the learning curve from junior as well as senior surgeons, to assess for failure to “complete” the learning curve, and analyze the impact of previous procedural experience on training data. Through an improved understanding of this learning pattern, we will safeguard excellence in training as technology evolves and maintain superior clinical outcomes while ensuring trainee surgeons are adequately equipped for independent clinical practice.

## Conflict of Interest Statement

The authors reported no conflicts of interest.

The *Journal* policy requires editors and reviewers to disclose conflicts of interest and to decline handling or reviewing manuscripts for which they may have a conflict of interest. The editors and reviewers of this article have no conflicts of interest.

## Figures and Tables

**Table E1:** Ovid search protocol

Line	Search
1	robot∗.mp.
2	surg∗.mp.
3	learning curve∗.mp.
4	RAT∗.mp.
5	RTS.mp.
6	1 and 2
7	4 or 5 or 6
8	3 and 7
9	(lobectomy∗ or lob∗ resect∗ or anatomical lung resection∗ or anatomical pulmonary resection∗ or segmentectomy or wedge resection∗ or pulmonary resection∗ or lung resection∗).mp.
10	8 and 9

**Table E2:** Study demographics

Study	Statistical method	Metrics measured	Surgeries included	Operating platform	d_1_	No. patients	No. surgeons	Conversions, %	Persistent air leak, %	Study duration, mo
Andersson, 2021^5^	CUSUM	Total operating time Inferior lobes operating time Superior lobes operating time	Lobectomy	Si	45	75	1	2.7	4	69
Arnold, 2019^6^	CUSUM	Total operating time	Lobectomy	Si	22	101	1	7.9	N/A	76
Cheufou, 2019^7^	Regression	Total operating time	Lobectomy	N/A (DV)	20	64	1		N/A	37
Fahim, 2017^8^	LOWESS, regression	Total operating time Total console time	Lobectomy Segmentectomy Wedge Bilobectomy	N/A	20	157	3	7.6	10.2	44
Feczko, 2019^9^	CUSUM	Total operating time 30-day mortality Major morbidity Perioperative transfusion	Lobectomy	N/A	20	1234, 1818, 2509	15, 21, 29	N/A	N/A	95
Fukui, 2021^10^	CUSUM	Total operating time Total console time Docking time	Lobectomy	Xi	28	79	5	0	N/A	47
Gallagher, 2018^11^	Regression	Total operating time	Lobectomy	Si	20	100	1	N/A	N/A	95
Igai, 2024^12^	CUSUM	Total operating time	Segmentectomy	Xi	16	30	1	0	N/A	40
Gómez-Hernández, 2012^13^	Line graph	Total operating time	Lobectomy	N/A	10	20	1	N/A	N/A	9
Kanzaki, 2021^14^	CUSUM	Total operating time	Lobectomy	Xi, S/Si (10)	10	68	1	N/A	4.4	77
Karnik, 2020^15^	Paired *t*-test, Fisher exact test	Total operating time	Lobectomy	Si	39	79	1	11.4	8.9	28
Le Gac, 2020^16^	CUSUM, Exponential modelˆ	Total operating time	Segmentectomy	Si	27, 31†	102	1	7	N/A	62
Lee, 2020^17^	Logarithmic regression	Total operating time	Lobectomy	Xi	20	118	1	N/A	9.3	27
Louie, 2012^18^	χ^2^ and Mann-Whitney *U*	Total operating time	Lobectomy Segmentectomy	N/A	6	46	4	N/A	4.3	29
Hernandez, 2022^19^	CUSUM	Total operating time Surgical failure	Lobectomy Bilobectomy Segmentectomy	N/A (DV)	17	75	1	4	1.3	25
Mazzella, 2023^20^	CUSUM	Total operating time Max heart rate Mean heart rate Mean resp rate	Lobectomy	Xi	28	72	1	2.8	8.3	17
Merritt, 2019^21^	Linear regression	Total operating time	Lobectomy	Xi	20	114	1	4.4	15.3	50
Meyer, 2012^22^	Regression	Total operating time Operative mortality Surgeon comfort	Lobectomy	N/A	15	185	2	N/A	3.2	95
Peng, 2020^23^	Line graph	Total operating time	Lobectomy Segmentectomy	Si	20	153	1	N/A	1.3	24
Song, 2019^24^	CUSUM	Total operating time Console time Docking time	Lobectomy	Si	32	252	1	N/A	2.4	24
Streit, 2024^25^	CUSUM	Total operating time	Lobectomy Bi lobectomy Segmentectomy	Xi	59, 44∗	195, 171∗	1	N/A	N/A	50, 33
Su, 2022^3^	CUSUM, Regression	Association with PAL	Lobectomy	Xi	50	305	4	3.6	8.3	32
Taniguchi, 2017^26^	χ^2^ and Mann-Whitney *U*	Total operating time	Lobectomy Segmentectomy	S/Si	20	44	1	N/A	N/A	67
Toker, 2016^27^	Regression	Total operating time Total docking Total console time	Lobectomy Segmentectomy Pneumonectomy	N/A (DV)	14	100	1	4	10	38
Veronesi, 2011^4^	Regression	Total operating time	Lobectomy	N/A (DV)	18	91	1	11	N/A	48
Xu, 2024^28^	CUSUM	Total operating time	Lobectomy Segmentectomy	Xi	40	400	1	0.8	N/A	22
Yang, 2023^29^	CUSUM	Total operating time Console time Surgical failure	Segmentectomy	Si/Xi	37	100	1	N/A	4	34
Yang, 2021 (Paper 2)^30^	CUSUM	Total operating time Console time Surgical failure	Lobectomy	Si	13	100	1	5	2.1	14
Zhang, 2019^41^	CUSUM	Total operating time Surgical failure	Segmentectomy	S	21	104	1	0	3.8	29

*CUSUM*, Cumulative sum; *N/A*, not available; *DV*, da Vinci mentioned, but no generation specified; *PAL*, prolonged air leak.

∗ Two sets of data offered for 2 surgeons.

† The unmarked 27 is the CUSUM calculation, and 31 is calculated by the exponential model.
